# Progressive Genomic Approaches to Explore Drought- and Salt-Induced Oxidative Stress Responses in Plants under Changing Climate

**DOI:** 10.3390/plants10091910

**Published:** 2021-09-14

**Authors:** Masum Billah, Shirin Aktar, Marian Brestic, Marek Zivcak, Abul Bashar Mohammad Khaldun, Md. Shalim Uddin, Shamim Ara Bagum, Xinghong Yang, Milan Skalicky, Teame Gereziher Mehari, Sagar Maitra, Akbar Hossain

**Affiliations:** 1 Institute of Cotton Research, Chinese Academy of Agricultural Sciences, Anyang 455000, China; kazimasum.agpstu20@gmail.com (M.B.); fiamieta21@gmail.com (T.G.M.); 2 Institute of Tea Research, Chinese Academy of Agricultural Sciences, South Meiling Road, Hangzhou 310008, China; aktershirin1992@gmail.com; 3 Department of Plant Physiology, Slovak University of Agriculture, Nitra, Tr. A. Hlinku 2, 949 01 Nitra, Slovakia; marek.zivcak@uniag.sk; 4 Department of Botany and Plant Physiology, Faculty of Agrobiology, Food, and Natural Resources, Czech University of Life Sciences Prague, Kamycka 129, 165 00 Prague, Czech Republic; skalicky@af.czu.cz; 5 Bangladesh Agricultural Research Institute, Gazipur 1701, Bangladesh; abkhaldun@gmail.com (A.B.M.K.); shalimuddin40@gmail.com (M.S.U.); happyshalim@yahoo.com (S.A.B.); 6 State Key Laboratory of Crop Biology, College of Life Sciences, Shandong Agricultural University, 61 Daizong St., Tai’an 271000, China; xhyang@sdau.edu.cn; 7 Department of Agronomy, Centurion University of Technology and Management, Village Alluri Nagar, R.Sitapur 761211, Odisha, India; sagar.maitra@cutm.ac.in; 8 Department of Agronomy, Bangladesh Wheat and Maize Research Institute, Dinajpur 5200, Bangladesh

**Keywords:** salt, drought, plants, ROS, genomics, approaches, integration

## Abstract

Drought and salinity are the major environmental abiotic stresses that negatively impact crop development and yield. To improve yields under abiotic stress conditions, drought- and salinity-tolerant crops are key to support world crop production and mitigate the demand of the growing world population. Nevertheless, plant responses to abiotic stresses are highly complex and controlled by networks of genetic and ecological factors that are the main targets of crop breeding programs. Several genomics strategies are employed to improve crop productivity under abiotic stress conditions, but traditional techniques are not sufficient to prevent stress-related losses in productivity. Within the last decade, modern genomics studies have advanced our capabilities of improving crop genetics, especially those traits relevant to abiotic stress management. This review provided updated and comprehensive knowledge concerning all possible combinations of advanced genomics tools and the gene regulatory network of reactive oxygen species homeostasis for the appropriate planning of future breeding programs, which will assist sustainable crop production under salinity and drought conditions.

## 1. Introduction

Global crop productivity is restricted due to abiotic stresses such as drought, salinity, flooding, nutrient deficiency, and environmental toxicity. Among these abiotic stresses, salinity and drought are the most severe constraints for sustainable agriculture on a global scale. Nearly 7% of terrestrial land is affected by salinity [1], while drought is widespread and increasingly common in recent years due to climate change. Altogether, salinity- and drought-affected lands cover approximately 10.5 and 60 million km^2^, respectively [2]. Furthermore, climatic changes worsen the frequency and intensity of water shortages in subtropical areas of Asia and Africa. As stated by the UN climatic report [http://www.solcomhouse.com/drought.htm; accessed date on 12 July 2021], rising temperatures are melting the Himalayan glaciers that feed Asia’s largest rivers (Indus, Ganges, Brahmaputra, Yangtze, Mekong, Salween, and Yellow), and those glaciers may disappear by 2035. Additionally, long-term trends indicate that the progressive proliferation of salinity has caused the dilapidation of arable land [3]. For instance, in California, over the last century, 4.5 out of 8.6 million hectares of wetted agricultural land have become salt-affected [4]. Currently, it has become a pertinent problem for crop production [5], mostly in arid and semiarid areas.

Based on numerous estimations, the world population will increase to over 9.7 billion by 2050, which will continue to exacerbate current global food insecurity issues. It is estimated that, over the past 50 years, improved crop productivity has brought about an increase in world food production by up to 20% per capita and decreased the proportion of food-insecure people existing in developing countries from 57% to 27% of the world population [6]. As a result, crops will need to cope with abiotic stresses such as drought and salinity and double productivity to further diminish food insecurity and support the growing human population in more ecologically sustainable ways.

Both drought and salinity stresses induce cellular dehydration, which causes osmotic stress, removal of water from the cytoplasm into the apoplast, and eventually evaporation into the atmosphere [2]. Moreover, early responses to salt stress and drought are comparable in plants. For example, plant cells prevent water loss by increasing the ionic constituents and decreasing the osmotic potential in stressed cells. Due to the similar mechanisms of the stress response in plants, it appears that drought and salinity tolerance mechanisms might be functionally interchangeable [7]. It is well known that stress response mechanisms involve several particular physiological and biochemical pathways that allow plants to adapt to unfavorable conditions. A number of abiotic stress factors, such as salinity, drought, high temperatures, and osmotic stresses, lead to the overproduction of reactive oxygen species (ROS), which cause serious cellular damage and hamper photosynthesis. To protect or repair these injuries, plant cells use an intricate defense system, including a number of antioxidative stress-related defense genes that, in turn, prompt changes in the biochemical plant machinery [8]. ROS production and antioxidant regulation all occur in a synergistic, additive, or antagonistic way and are associated with the control of oxidative stress.

**Figure 1:**
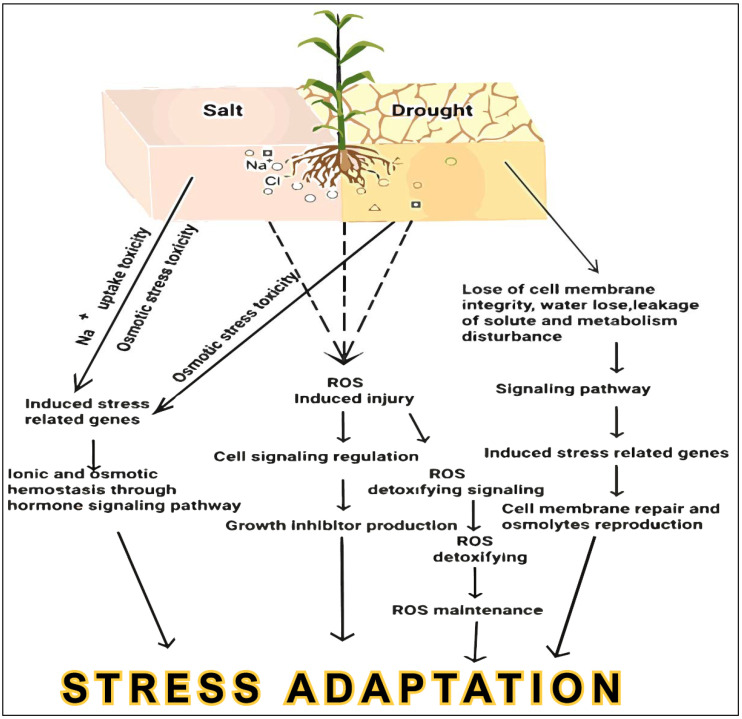
The mechanisms involved in crop salt and drought stress responses.

Nevertheless, plant stress response mechanisms are controlled by convoluted networks that are determined by environmental and genetic factors that are often difficult to untangle, thereby impeding traditional breeding approaches [9]. Considering that the conventional breeding strategies for crop improvement are largely aimed at improving yield to meet the demands of an ever-growing world population, breeders have to implement innovative approaches in agriculture to combine high-yield and abiotic stress-tolerant traits in crops [10]. Recent scientific advances and the abovementioned challenges in agriculture have directed the development of high-throughput techniques to pursue and take advantage of plant genome research for the improvement of stress-tolerant crops. Thus, these genomics approaches focus on the entire genome, involving genic and intergenic positions, to attain new insights into the functional and molecular responses of plants, which will sequentially offer specific techniques for crop plant improvement. Recently, many scientists have revealed promising outcomes toward understanding the molecular mechanisms of abiotic stress tolerance in prospective crops using progressive molecular biology practices [10,11,12,13,14,15,16,17,18,19,20,21]. The mechanisms involved in crop salt and drought stress responses are discussed in Figure 1. In this review, we described in detail how to mine the functional genes involved in drought and salt response in plants, using methods such as traditional QTL, transcriptomic analysis, and GWAS. Then, we explored approaches such as epigenetic regulators, gain-of-function, RNAi, TALENs, ZFNs, CRISPR, base editing, and primer editing for functional verification with an example of target genes generated by the aforementioned approaches. Finally, we summarized the methods for generating salt and drought-tolerant crops. The review aimed to offer comprehensive knowledge for improving salt- and drought-tolerant crops using modern genomics strategies regarding ROS regulatory networks. The overview of earlier studies on the advancement of genomics approaches will help in the investigation of upcoming research instructions for improving salt- and drought-tolerant crops.

## 2. Mining Approaches for Salt and Drought Stress Response Genes

To improve drought and salinity stress tolerance in crops, we first require comprehensive knowledge concerning the complex mechanisms of plants that respond to stresses. Detecting the genes/markers/QTL regions associated with drought and salinity stress responses is the first crucial step toward reaching the required understanding for breeding drought and salinity stress-tolerant crop varieties. For the discovery of a gene, various strategies are available in both model and nonmodel crops; here, some of the most advanced are discussed briefly.

### 2.1. Quantitative Trait Loci (QTL) Analysis

A quantitative trait locus (QTL) is a gene or a region of DNA that is associated with the variation of a quantitative/phenotypic trait that must be polymorphic to affect the biological population. QTL mapping has been a powerful tool for dissecting genetic variants underlying quantitative traits in numerous biological studies and breeding programs. There are two primary concerns when using QTL mapping. One is the power for QTL identification under a controlled false-positive rate, and the other is the accuracy of QTL localization [22]. QTL mapping has been applied as a technique for identifying genomic regions significantly correlated with grain output and various genetically intricate characteristics in cereal crops. This technique is particularly powerful when genetic variation is studied concerning numerous complex traits, where it is possible to identify and differentiate genomic regions that contribute to different characteristics of interest. The data relevant to QTL mapping can be conducive to enhancing the genetic potential of crops via marker-assisted breeding [23]. Currently, scientists can link the molecular mechanisms of genes found in QTLs to demonstrate the genetic and physiological basis of traits such as grain yield. A nice example of this cooperation of QTL mapping, trait scoring, and breeding can be found in using green coloration as a metric of drought resistance in sorghum. The genetic dissection of molecular QTLs associated with green coloration during drought lends convenience to demonstrate the basic mechanisms of physiology and investigate the molecular causes of drought tolerance in sorghum and different grasses [24]. Reducing the genomic sizes of QTLs facilitates enhanced targeting of pertinent genomic regions. Improving the fine mapping of QTLs improves the efficiency with which breeders can understand the significance and mechanisms of QTLs relevant to their traits of interest [24]. Enhanced QTL mapping is particularly relevant when deconvolving complex genomic regions. For example, hypostasis of alleles within QTLs, QTL-QTL genetic interactions, context-dependent activities of QTLs, and the QTL marker position itself impact the articulation of a complex trait such as the yield of grain under drought stress [25]. Interestingly, fine mapping of QTLs revealed that an individual main QTL controlling membrane potential vastly improved marker-assisted selection for salinity-stressed barley [26]. Thus, fine QTL mapping is required for marker-aided QTL pyramiding to improve drought tolerance [27]. Identification of QTLs for abiotic stress tolerance suggests augmentations that can be used for further genomics studies toward the detection of noble genes of salt and drought tolerance to develop a new variety [28]. Several examples of QTLs (quantitative trait loci) for improving crop plant production under salinity and drought stresses are discussed in Table 1.

### 2.2. Forward Genetics and the Candidate Gene Strategy

Crop plants with stress tolerance have been generated by the transference of genes/loci from definite donor parents, either through a forwarding genetics method that includes the determination of a gene function linked with a phenotype or the identification of novel stress-tolerant donor lines created by the use of mutagenesis. In contrast, reverse genetic breeding approaches could offer an understanding of gene functions and structure/sequence information to predict traits for adapting stress-tolerant cultivars using transgenic and advanced breeding tools. In genomic studies, researchers have implemented these approaches for the genetic improvement of various model and nonmodel species toward salt and drought stress tolerance [34,35,36,37,38,39,40,41].

### 2.3. Transcriptomics Analysis

Transcriptome analysis refers to the study of the transcriptome of the entire set of RNA transcripts that are generated by a genome, under given times and circumstances or in a specific cell. Transcriptomic analysis techniques play a crucial role in the identification of candidate gene functions and pathways that respond to specific environments [42]. In the last decade, universal transcriptome analysis approaches have been particularly advantageous for functional genomic studies that offer comprehensive molecular mechanisms of certain phenomena. Primarily, a global transcriptome study was initiated with suppression subtractive hybridization (SSH) and cDNA-AFLP and acquired a quantum dive to RNA-seq with the progression of NGS platforms [10]. The information content of an organism is held in its genome and articulated through transcription. The basic purposes of transcriptomics are to record the transcription of all species, including mRNAs, noncoding RNAs, and small RNAs; to determine the transcriptional configuration of genes in terms of their start sites, 5′ and 3′ ends, splice variants, and other posttranscriptional modifications; and to calculate the varying expression patterns of every transcript throughout development and under diverse conditions [40,41]. Currently, transcriptome profiling has progressed into nearly all organisms and represents how information attained from sequence data can be converted into a wide knowledge of gene functionality [42]. Plant stress-response mechanisms frequently employ the use of transcription factors (TFs). A TF is a protein that targets, typically, multiple genes that comprise a regulon and influences their expression patterns. Thus, TFs are a powerful tool for the genetic regulation of many downstream genes and processes, including abiotic stress responses [43]. In the case of salt and drought stress, transcripts related to the upregulation of vital biochemical pathways required for cellular osmotic balance, abscisic acid, and cellular water uptake are controlled by TFs [44]. The role and example of various transcription families through transcriptome analysis relevant to salt and drought stress tolerance are discussed in Table 2.

### 2.4. Association Mapping

In genetics studies, association mapping (also well known as linkage disequilibrium mapping) refers to the regular genome-wide distribution of several genes together with other measurable loci (markers) in predicting marker-trait relatives [52] applied in various crops, including rice, barley, maize, sorghum, and wheat, to identify the significant genes or markers that confer a given trait [53]. Numerous genes have been indicated as being connected with abiotic resistance by applying association mapping [54]. It has also been applied to inquire about the limitations within focused parameters and molecular markers in different crops [55]. Association mapping is extremely efficient in experimental varieties with complex or unknown genotypes or those that have a large regeneration time. Association mapping of drought-related varieties in barley was applied to terminate a conventional biparental system of QTL mapping [56]. Furthermore, association mapping has been used to progress the development of QTL maps [57]. A detailed discussion on the association mapping for the sustainability of crop production under salinity and drought stresses is available in Table 3.

### 2.5. Genome-Wide Association

**Figure 2:**
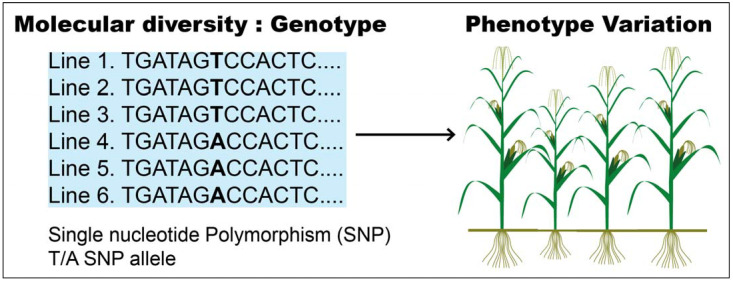
Connection of genotype to phenotype variation.

GWAS (genome-wide association study) is a potent presumption-free method used to identify and dissect the genetic regions associated with a certain trait. Typically, GWAS is performed by scoring the phenotypes and sequencing many individuals to link genotype to phenotype, thereby linking genetic variants to a given trait (Figure 2) [61]. 

GWAS applies large markers and several populations of non-cross-executed lines to provide larger mapping exploration than traditional QTL mapping based on a cross-evolved segregating population, leading to the detection of unknown or unexpected genes. It has been applied to separate complicated genetic parameters in leading crops such as rice and wheat under salt and drought stress. Additionally, GWAS has been effectively conducted to designate QTLs for particular characteristics in wheat (e.g., grain yield, morphology relevant to leaf rust disease, and end-usage quality), thereby applying various systems of molecular markers to bolster breeding resources [62,63]. GWAS has detected more than 2000 loci for simple human diseases to date [64]. Therefore, compared with QTL mapping, GWAS delivers an in-depth, cost-efficient mode of gene investigation and detection of molecular markers.

GWAS focused on the flowering period of saline-treated rice identified 11 loci bearing 22 important SNPs linked to stress responses. The potential genetic determinant of germination was identified on chromosome one, close to the saline conditional QTL regulating Na^+^ and K^+^ levels. Approximately 1200 candidate genes linking development to sodium and potassium ion allowances were detected [65]. Thus, GWAS offered an informed list of candidates for saline tolerance-connected gene cloning and uncovered responsive genetic elements relevant to salt stress [66]. GWAS is also important to perceive the genetic architecture of complex characteristics to improve drought tolerance [67]. Recently, “No-Genome-Required-GWAS” approaches have provided easy and efficient identification of genetic variants underlying phenotypic variation in plants [68]. Details on genome-wide association mapping for identifying QTLs under salinity and drought stresses are discussed in Table 4.

### 2.6. Next-Generation Sequencing

Sequencing technologies include several techniques that generally consist of template preparation, sequencing and imaging, and data analysis [79]. Next-generation sequencing (NGS) integrates technologies that inexpensively and efficiently produce millions of short DNA sequence reads mainly in the range of 25 to 700 bp in length [80]. These technologies have made it possible for scientists to investigate crops at the genomic and transcriptomic levels to assist diversity analysis and marker-assisted breeding [80]. The relevance of NGS appears to be endless, permitting quick presses forward in numerous fields associated with the biological sciences. NGS has also afforded a wealth of knowledge for biology studies via end-to-end whole-genome sequencing of a broad diversity of organisms [81]. Whole-genome sequence studies have focused particularly on detailed information on genomics criteria, including regulatory sequences, coding and noncoding genes, GC content, and repetitive elements, which would be utilized in functional characterization, such as microarray or tiling arrays. Additionally, NGS can be used to address many remaining biological questions by means of resequencing targeted areas of concern or whole genomes (as is being performed for the human genome [82]), *de novo* assemblies of bacterial and lower eukaryotic genomes, cataloging the transcriptomes of cells, tissues, and organisms (RNA sequencing), genome-wide profiling of epigenetic markers and chromatin structure using additional seq-based methods (ChIP-seq, methyl-seq, and DNase-seq), and species classification and/or gene discovery by metagenomics studies [50].

## 3. Functional Genomics Approaches

After identifying a QTL/allele/gene, the next sensible step is to characterize the gene before incorporation into a cultivar by studying several physiological, molecular, and biochemical pathways of genes. Thus, functional genomics approaches were extensively implemented to determine the gene functions and the connections between genes in a regulatory network that would be utilized to produce improved crop varieties. Consequently, there have been multiple tools developed for the characterization of gene function; some of the most exploited are described briefly.

### 3.1. Epigenetic Regulators

In wider definitions, the term ‘epigenetics’ frequently refers to a type of overall nongenetic (unrelated to DNA sequence *per se*) heredity at various levels. That is, epigenetics illustrates a number of dissimilar methods of genetic regulation whose temporal and heritable constituents have not in all cases been decided [83]. For example, methylation of DNA generally interferes with gene expression by way of gene silencing [84]. The reduction of methylation in resistance-associated genes activates chromatin and the expression of genes, which offers long-term or enduring resistance under stress conditions [85]. Epigenetics sustains the identity of stress memory in plants, which helps pre-exposed plants fight comparable stress throughout subsequent exposures. Histone modifications, DNA methylation and demethylation, and ATP-dependent chromatin remodeling are some of the epigenetic changes performed by plants during drought stress [86]. Epigenetic responses to drought stress have been studied in numerous plants, particularly the stress memory and gene activation marker *H3K4me3*, which has been used to carry out genome-wide ChIPseq analyses in Arabidopsis [87]. Furthermore, the HAT genes in rice (*OsHAC703, OsHAG703, OsHAF701*, and *OsHAM70*) [88] and the *HvMYST* and *HvELP3* genes in barley were also shown to be involved in epigenetic regulation in drought responses [89]. DNA methylation and histone modifications may have a similar result on stress-inducible genes, as salinity stress influences the expression of a range of transcripts in soybean [48]. Work in rice underlined that hypomethylation in reaction to salt stress may be associated with changes in the expression of DNA demethylases [90]. The transcriptional adaptor ADA2b (a modulator of histone acetyltransferase activity) is responsible for hypersensitivity to salt stress in *Arabidopsis thaliana* [91].

### 3.2. Gain-of-Function Lines

Gain-of-function methods have been extensively used for the study of gene function in plants and are considered among the most useful tools for gain-of-function phenotypes. Gain-of-function lines are generated through the arbitrary activation of endogenous genes by transcriptional enhancers and the regular expression of individual transgenes by transformation [9]. This method employs the phenotype of gain-of-function lines that overexpress a selected gene family and can be executed without meddling from other gene family members that allow the categorization of functionally unwanted genes [10]. Alternatively, the overexpression of a mutant gene can be expressed due to the presence of higher levels of nonfunctional protein causing a superseding negative interface with the wild-type protein. To overcome this event, a mutant type could be used to compare the wild-type protein allies, resulting in a mutant phenotype. Conversely, heterologous expression of a gene in the yeast-hybrid system is an alternative way to characterize genes. In the first gain-of-function approach, a strong promoter or enhancer element is arbitrarily inserted into the plant genome with the help of T-DNA [11], which stimulates a gene near the site of the harbor. Other gain-of-function approaches involve cDNA overexpression and open reading frame (ORF) overexpression, whereas full-length cDNAs or ORFs have been cloned into a strong promoter downstream. Under the switch of the CaMV35S promoter, various abiotic stress response genes have been characterized by the use of ectopic overexpression of cDNAs [11,12,13,14].

### 3.3. Gene Silencing and RNA Interference Techniques for Salinity and Drought Stress

Suppression of a gene is referred to as gene silencing in plants and fungi and interference RNA (RNAi) in animals and is generally thought of as a controlling mechanism of gene expression mostly in eukaryotic cells [92]. RNA interference (RNAi) has been considered one of the most crucial discoveries in molecular genetics during the last several years for posttranscriptional gene silencing (PTGS) cosuppression [93]. RNA silencing hints at a nucleotide sequence-specific procedure that prompts mRNA degradation or translation termination at the posttranscriptional level in plants arbitrated by small RNAs (sRNAs), which are divided into two classes: microRNAs (miRNAs) and small interfering RNAs (siRNAs). However, RNAi was properly adapted into antisense-stranded RNA as an operative technique to constrain gene expression [94]. Silencing a gene through transgenic expression of sRNAs has been extensively implemented for abiotic stress-related gene function functional efforts. Currently, the virus-induced gene silencing (VIGS) technique for posttranscriptional gene silencing is extensively used for rapid and efficient gene function studies related to salt and drought stresses [95,96,97,98]. It can also be used for both forward and reverse genetic studies. Target gene silencing techniques for improving crops under salinity and drought stresses are discussed in Table 5.

### 3.4. Genome Engineering (TALENs, ZFNs, CRISPR/Cas)

**Figure 3:**
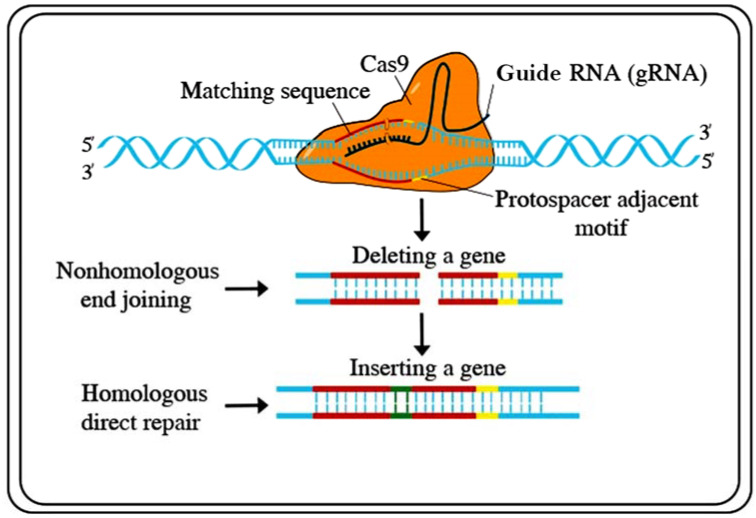
CRISPR/Cas9 is a powerful tool for genome editing of Cas9 to a guide RNA that directs the complex to a place on the DNA double helix and contains the code for the addition of a new DNA sequence at the double-stranded break. Source: adapted and modified from [www.stockadobe.com; accessed date on 12 July 2021].

Recently, several functional genomics-based strategies have been developed for genetic engineering. To improve crops for sustainable food production, targeted genome engineering has become a substitute for conventional plant breeding and transgenic (GMO) strategies, including transcription regulators, epigenetic modifiers, DNA integrators, TAL effector nucleases (TALENs), zinc-finger nucleases (ZFNs), clustered regularly interspaced short palindromic repeats (CRISPR)/Cas (CRISPR-associated proteins), and base editors and prime editors. Until recently, the existing methods have been considered to be unwieldy. Both TALENs and ZFNs could be used to mutagenize genomes at exact loci. However, the problem is that these systems need two altered DNA-binding proteins flanking a sequence of interest, each with a C-end FokI nuclease unit [106]. For plant research, these techniques have not been extensively implemented. Recently, a technique based on the bacterial clustered regularly interspaced short palindromic repeats (CRISPR)/Cas (CRISPR-associated proteins) type-2 prokaryotic adaptive invulnerable system has been developed as an alternate process for genome engineering [106]. The CRISPR/Cas (clustered regularly interspaced short palindromic repeats/CRISPR-associated proteins) system was first identified in bacteria and archaea and can cleave exogenous DNA substrates [107]. CRISPR/Cas has since been modified to be used as a gene-editing technology. However, CRISPR/Cas9 has largely overtaken the other aforementioned gene editing practices. Investigators express similar stories: a few years ago, they started working on projects using both TALENs and CRISPR/Cas9 side-by-side but rapidly established CRISPR systems [108]. Graphical presentations of the CRISPR/Cas9 techniques are available in Figure 3.

The beginning of CRISPR has made it conceivable to rewrite host DNA by introducing some major amendments. These modifications include gene replacement, deletions, inversion, knockouts, and translocations [109]. Using CRISPR/Cas9 tools, several genes, such as OsERF922, OsPDS, OsERF922, ERFs, OsHAK1, Badh2, OsRR22, and TMS5, were knocked out, and a predictable phenotype was attained [110,111,112,113,114,115,116]. More promising are the potential forecasts of this technique for producing plants with specifically tailored purposes, i.e., biofuel production, synthetic biology, disease resistance, phytoremediation, etc. [117]. This technique also offers a new method for abiotic stress breeding programs [113]. Several examples of CRISPR/Cas9 technology-mediated improvements to plant tolerance to abiotic stress are discussed in Table 6.

### 3.5. CRISPR-Mediated Base Editing and Prime Genome Editor

**Figure 4:**
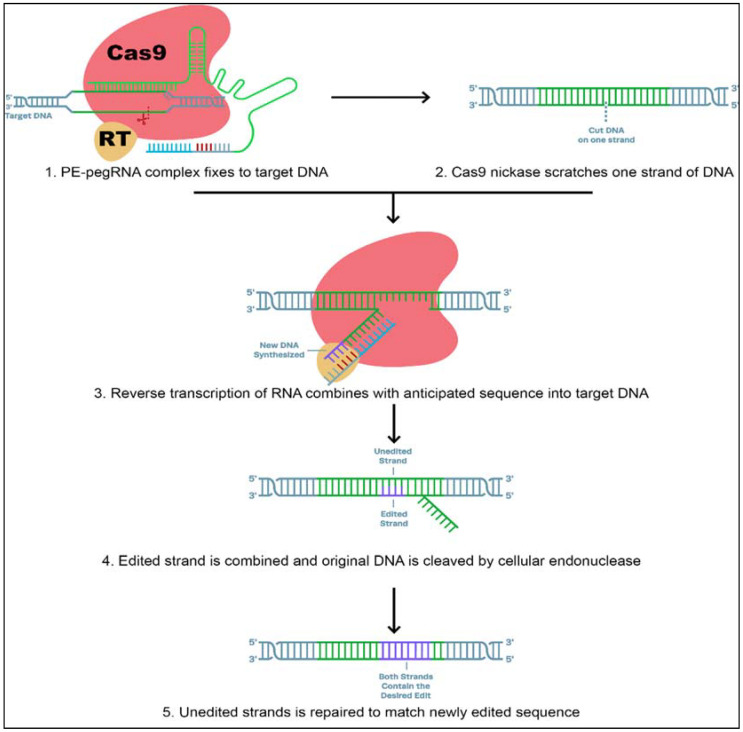
A new tool (prime editor) of DNA manipulation that couples two enzymes, Cas9 (brown) and reverse transcriptase (yellow), to a guide RNA (red) that directs the complex to an exact place on the DNA double helix and contains the code for the addition of a new DNA sequence at the double-stranded break. [Figure modified from: https://www.synthego.com/guide/crispr-methods/prime-editing; accessed date on 12 July 2021].

It is well known that CRISPR is a powerful genome-editing technique. CRISPR can change genes and edit DNA sequences by producing double-strand breaks in double-helical DNA, leaving the cell to repair the breakage (Figure 4). 

The control mechanisms over the repair process are the main limitations in basic research and plant sciences. However, several groups recently reported the “base editing” system, a new approach for site-directed mutagenesis of genomic DNA. Base editing tools are highly efficient, reduce the rate of off-target effects, and do not require DNA double-strand cleavage or donor template repair. These methods make use of a Cas9 nickase fused to various deaminases. Specific C-to-T or A-to-G transitions in genomic DNA are catalyzed by these fusion proteins. The base editor and Target-AID (target-activation-induced cytidine deaminase) systems are two representative architectures of cytidine base [127,128]. Therefore, engineering of single-plasmid CRISPR-mediated base editing tools for *S. meliloti* that included adenosine base editors (ABEs), cytidine base editors (CBEs), and glycosylase base editors (GBEs) is capable of achieving both base transitions (A-to-G, C-to-T) and transversions (C-to-G) [129]. Base editing has become a widely applicable tool for gene disruption in a variety of bacteria [17,22,28,130]. Nevertheless, the new invention “prime editor” makes the successful addition or deletion of exact sequences within the genome possible with minimum off-target effects [129].

The creators claim that their tools can precisely target approximately 89% of recognized pathogenic human genetic variants. Prime editing may have fewer bystander mutations than base editing, especially when multiple Cs or As are present in the editing activity window [131]. It is also less constrained by the availability of protospacer adjacent motif (PAM) than other methods such as homology directed repair (HDR), non-homologous end joining (NHEJ), or base editing, because the PAM-to-edit distance can be greater than 30 bp on average [26]. Nevertheless, there is a large suite of base editors that have been developed with improved efficiency, product purity, and DNA specificity, as well as broad applicability [25]. Although prime editing has the potential to replace base editors, the technology is still in its early stages and is typically less efficient than current generation base-editing systems with superior on and off-target DNA editing profiles [20]. Consequently, a suitable editing strategy for specific applications must be chosen based on various criteria for gene-editing, such as the desired edit, the availability of PAMs, the efficiency of editing, and off-target/bystander mutations. 

## 4. The Development of Salt- and Drought-Tolerant Crops with High Yielding Capacity

The generation of crop varieties with a high level of tolerance to salinity and drought is vital for creating full yield potential and sustainable production. Generally, there are two methods to integrate enhanced traits such as drought and salinity stresses in plants: genetic engineering and breeding programs.

### 4.1. Genetics Engineering

The advent of modern genetic engineering strategies offers the generation of plants with rising abiotic stress tolerance. Under abiotic stress conditions, several genes of crop plants in different pathways lead to upregulation of expression. Stress-responsive genes and their controlling genes can be transferred and expressed in different species using an Agrobacterium-mediated transformation system involving molecular, biochemical, and physiological changes that direct an increase in plant growth, development, and yield under stress environments [16]. Currently, the use of stress-inducible promoters for the expression of stress response genes has confirmed a time-specific and optimal level of expression. Salinity and drought are major environmental stresses that adversely affect the growth and development of crops; thus, a number of genes encoding proteins involved in the biosynthesis of stress defensive elements, including glycine betaine, mannitol, and heat shock proteins, have been used for abiotic stress tolerance, as well as several transcription factors, such as MAPK, bZIP, AP2/EREBP, WRKY, and DREB1 [17,18]. However, overexpressed transgenes can function as positive regulators of tolerance to a single stress or multiple stresses, such as salinity, drought or both. Therefore, the newly developed transgenic plant might have to be tolerant to single or multiple stresses, have high yields, and be devoid of harmful pleiotropic traits. Posttranslational modifications, orthologous gene expression of effectors from wild relatives or halophytes, gene expression by regulating miRNA activity, osmoprotectants, gene pyramiding, engineering of transcription factors, chaperones, late embryogenesis, metabolic pathways, abundant proteins, epigenetics, and even chaperones have been implemented to produce a new generation of transgenic plants [130]. Successful salinity- and drought-tolerant transgenic crops were produced and approved for cultivation as food and feed [23,27,28,29].

### 4.2. Gene Introgression

Introgressiomics is designated as an extensive systematic improvement of plant genomes and populations through bearing introgressions of genomic fragments from wild crop relatives relative to the genetic background of established crops to develop new cultivars with promising traits [24]. Through introgression, greater genomic plasticity can be attained in a crop using exotic genetic material that was previously nonexistent within the genome [104]. For crop improvement, genetic engineering strategies are relatively faster than traditional breeding programs, as well as cloning of genes responsible for imperative traits and introgression into plants [104]. To develop salt- and drought-tolerant varieties, a particular breeding program can be established through an understanding of the physiological and genetic mechanisms of these stresses. MAS improves the speed and efficacy of breeding because genetic markers are unaffected by the environment, are efficient to use in early generations [105], and can be useful for the introgression of target genes. Successful stories of introgression in various crops for many traits, including both abiotic and biotic stress tolerance/resistance, have been implicated from wild relatives in cultivation without affecting yield and quality [24,106,107].

### 4.3. Marker-Assisted Breeding and Transference of Genes

Marker-assisted breeding is a process that permits breeders to track traits over generations of breeding using genetic markers associated with a given trait. In marker-assisted breeding, DNA markers associated with desirable traits are used to identify and choose plants containing the genetic locus that confers the desirable trait. DNA markers have a high probability of increasing the capability and accuracy of traditional plant breeding via marker-assisted selection (MAS). MAS allows for quicker and more efficient selection of desired crops, as cultivators can reliably test for the presence of a genetic marker associated with a trait rather than waiting to assess the trait itself. The most efficient and extensively applied method for MAS is marker-assisted backcrossing [132]. Marker-assisted breeding is in contrast to the direct addition of a gene or multiple genes to enhance a trait, such as genetic modification. Using genetic markers in breeding depends on the phenological acclimatization of the acceptor genotype, and the introduction of a new marker or allele may be necessary to increase the yield. With the advent of molecular markers and MAS technology, numerous studies have capitalized on such technology to identify genes or QTLs affecting sequence tagging in different plant species during different developmental stages, to identify genes or QTLs that were introduced into different plant varieties, and to gain an overall deeper and more efficient understanding of QTLs that contribute to complex traits [133]. Marker-assisted breeding for improving crop quality under salinity and drought stresses is discussed in Table 7.

**Figure 5:**
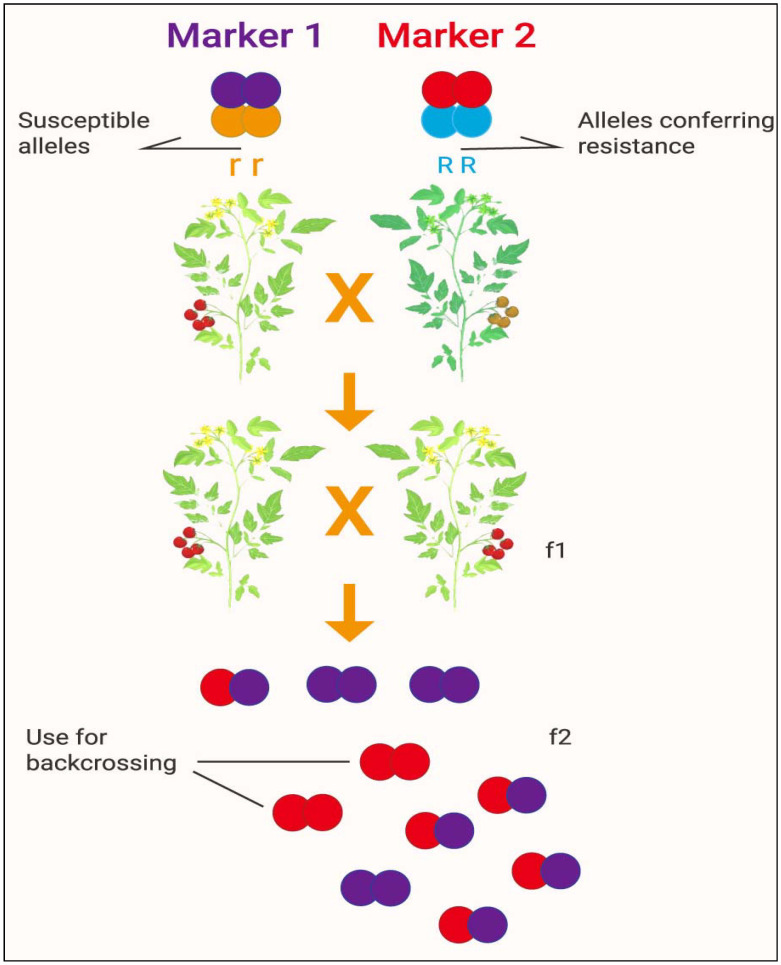
Development of a new crop variety by marker-assisted selection. Source: modified from: [http://b4fa.org/bioscience-in-brief/plantbreeding/how-do-you-develop-a-new-crop-variety-by-marker-assisted-selection-mas/; accessed date on 12 July 2021]. Note: Marker 1 and Marker 2 confer susceptible and resistance alleles, respectively; f1 and f2 indicate the first and second filial generations of offspring, respectively.

Current advances in genomics and genome sequencing in rice have made it feasible to locate and precisely map a certain number of genes via linkage to DNA markers. MAS can be applied to control the presence or absence of genes and has also been applied to assess the contributions of such genes conferring traits that have been introduced into extensively developed varieties [26]. Coupling genomic resources with the utility of MAS, breeders can now gain unprecedented insight into the genetic regulation of complex traits. MAS is a large advantage for developing new crop varieties because crops with ineligible gene aggregations can be dispelled from the selection process. This offers breeders the opportunity to focus on a reduced number of candidate lines for breeding targets in successive generations [131]. It has been shown that association mapping along with population formation and screening of cotton germplasm can improve QTL assignment and MAS [131]. Combining MAS and GS (genomic selection) with adequate genetic variety, databases, analytical instruments, and well-established climate and soil data is a powerful way to produce modern varieties with high drought resistance that can be readily inaugurated into appropriate agricultural programs [27]. These methods could produce a high number of lines of a crop appropriate for propagating crops in a range of drought and salinity stress ecosystems. Furthermore, incorporating these data can lead to the creation of varieties that can be further optimized to control largely heritable principal secondary characteristics. MAS delivers precise, rapid, and profitable progress toward the development of crop varieties that can be applied to abiotic stress tolerance [26]. A graphical presentation of the development of a new crop variety by marker-assisted selection is available in Figure 5.

## 5. Involvement of Genes in the Regulation of ROS in Abiotic Stress Tolerance

**Figure 6:**
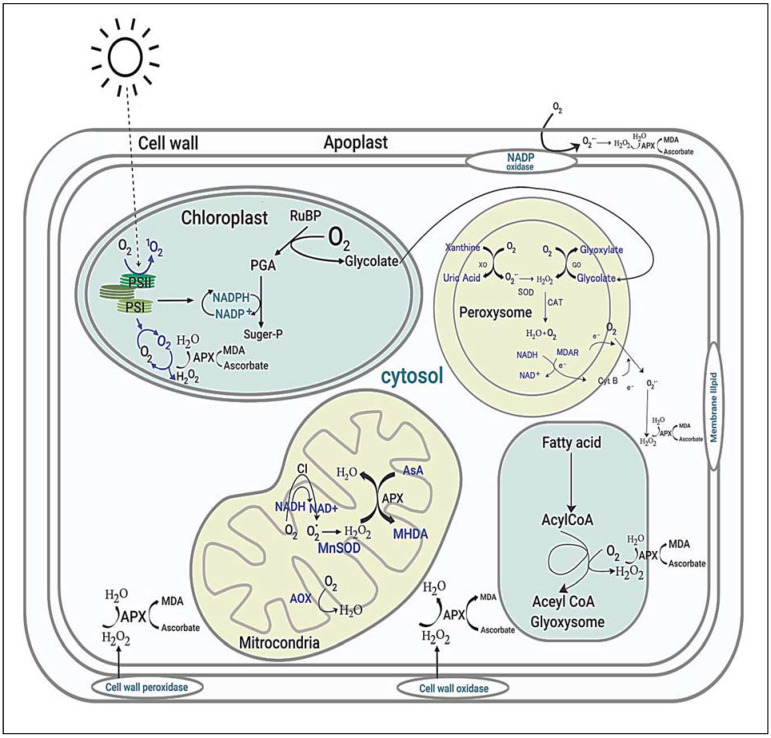
Sites and typical regulation of ROS in plant cells. PSII; Photosystem II, PSI; Photosystem I, MDA; Malondialdehyde, APX; Ascorbate Peroxidase, PGA; 3-Phosphoglyceric acid, H_2_O_2_; Hydrogen peroxide, SOD; Superoxide dismutases, CAT; Catalase, MDAR; Monodehydroascorbate reductase, NADH; Nicotinamide adenine dinucleotide, Alternative oxidase.

Reactive oxygen species (ROS) are assumed to play roles in many noteworthy signaling reactions in plant metabolism. Under drought and salinity environments, interrupting photosynthesis and increasing photorespiration intermittently alter the regular homeostasis of cells and influence the production of ROS in mitochondria, chloroplasts, and peroxisomes (Figure 6) [142,143].

**Figure 7:**
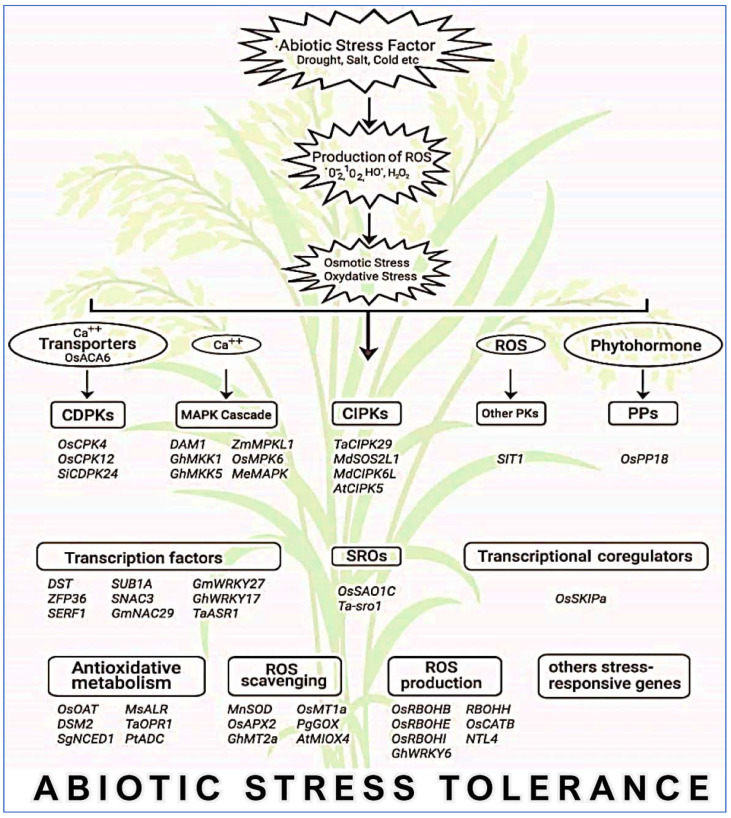
A general view of major genes that are intricate in abiotic stress resistance through ROS maintenance in crops. MAPK, mitogen-activated protein kinase; CDPK, calcium-dependent protein kinase; CIPK, calcineurin B-like protein-interacting protein kinase; PK, protein kinase; PP, protein phosphatase; SRO, similar to RCD.

In addition to organelles, the plasma membrane together with the apoplast is the main site for ROS production in response to endogenous signals and exogenous environmental stimuli [144]. Overproduction of ROS in plant cells is extremely reactive and noxious to proteins, lipids, and nucleic acids, which finally results in cellular damage and death initiated by stressful environments [142]. ROS-scavenging enzymatic antioxidants (SOD, APX, CAT, GPX, MDHAR, DHAR, GR, GST, and PRX) and nonenzymatic antioxidants (GSH, AsA, carotenoids, tocopherols, and flavonoids) are located in different sites of plant cells, and they directly or indirectly play a key role in ROS homeostasis via different unique pathways to avoid oxidative damage. In addition, soluble sugars as well as disaccharides, raffinose family oligosaccharides, and fructans play a dual role in ROS maintenance [145]. Consequently, crop plants have executed several interrelated signaling pathways to operate different groups of genes (Figure 7), which are induced under stress conditions to generate different classes of proteins, for example, protein kinases, enzymes, transcription factors, molecular chaperones, and other efficient proteins, subsequent to various physiological and metabolic reactions to improve tolerance to multiple environmental stresses.

It is well known that antioxidants stimulate gene expression linked with responses to various environmental signals to exploit protection through the regulation of cellular ROS levels and redox state [146]. The characteristics and roles of selected genes and their processes under salinity and drought stresses are discussed in detail in Table 8 and Table 9.

## 6. Conclusions

The adverse effects of climatic change and an increasing population pose a momentous challenge to crop production and food security, particularly in developing countries. Thus, it is a prerequisite to understand plant response mechanisms to abiotic stresses, namely, salinity and drought, at the molecular level to improve crop productivity. To overcome these circumstances, conventional breeding systems are no longer appropriate avenues to bolster crop production. In this review, we mainly discussed advanced molecular genomics tools focusing on plant genes in response to abiotic stress mechanisms to update our knowledge on the rapid development of high-yielding crop varieties under salt and drought stresses. Moreover, we summarized the recent studies of plant genes and differentiated them according to their molecular functions in response to salt and drought and reported recent advances in these stress-response mechanisms. Finally, the integration of any two or all three genomics approaches would be used to generate salinity- and drought-tolerant crops.

## Figures and Tables

**Table 1:** Known QTLs (quantitative trait loci) for improving crop plant production under salinity and drought stresses.

Stresses	Crops	Major Effect/Finding	References
Drought stress	Cowpea	Detected QTL relevant to salt-tolerant and sensitive varieties	[29]
Drought stress	Wheat	Detected genetic loci to major morpho-physiological traits, components of yield, and grain yield	[30]
Salinity	Barley	Detected QTLs related to stomatal and photosynthetic traits associated with salinity tolerance	[31]
Drought	Sorghum	Identified QTLs associated with flowering and drought resistance	[24]
Drought	Rice	Improved crop yield under drought tolerance	[25]
Drought and submergence tolerance	Rice (TDK1)	Drought and submergence tolerance and yield stability	[25]
Drought and flood	Rice	Detected drought and salinity tolerance varieties based on developmental and physiological traits	[32]
Salinity	Rice (Pusa Basmati 1121)	Detected two QTLs for drought and one QTL for salt stress	[32]
Drought	Upland rice	Identified QTLs relevant to leaf rolling, leaf drying, leaf relative water content, and relative growth rate under water stress	[33]

**Table 2:** Identification of different TF families through transcriptome analysis relevant to salt and drought stress tolerance.

SL No.	TF Family	Gene ID	Crop Variety	Target Stresses	References
1	AP2/EREBP	*TaERF3*	*Triticum aestivum*	Drought, Salt	[45]
2	bZIP	*GmbZIP1,*	*Glycine max*	Drought, Salinity,	[46]
3	MYB/MYC	*StMYB1R*-*1*	*Solanum tuberosum*	Drought	[47]
4	NAC	*OsNAC5, GmNAC20*	*Oryza sativa, Glycine max*	Drought	[48,49]
5	WRKY	*TaWRKY44*	*Triticum aestivum*	Drought, Salt	[50]
6	*AREB/ABF*	*AREB1, AREB2/ABF4* and *ABF3*	*Arabidopsis thaliana,*	Drought	[51]

**Table 3:** Association mapping for improving crop production under salinity and drought stresses.

Stresses	Crops	Target Gene	Major Findings	References
Salinity	Cowpea (*Vigna unguiculata* (L.)		Association mapping for salt tolerance at germination and seedling stages and the identification of SNP markers associated with salt tolerance in cowpea	[58]
Drought	Wheat	*RM223*	Demonstrated a strong power of joint association analysis and linkage mapping for the identification of important drought response genes in wheat	[59]
Salinity	Cotton (*Gossypium hirsutum* L.)		Provided reference data for the use of MAS for salt tolerance in cotton	[55]
Salinity	Cotton *(Gossypium arboretum)*	*(Cotton_A_37775* and *Cotton_A_35901)*	Provided fundamental information to produce novel salt-tolerant cultivars	[54]
Drought	*Pearl Millet*	*PMiGAP*	Development of high-yielding drought- and submergence-tolerant rice varieties using marker-assisted introgression	[60]

**Table 4:** Genome-wide association mapping for identifying QTLs under salinity and drought stresses.

Stresses	Crop Variety	Major Effect/Finding	References
Heat prone	*Spring wheat*	Yield stability	[69]
Drought	Rice (indica and japonica)	Identified QTL containing promising candidate genes related to drought tolerance by osmotic stress adjustment	[70]
Salt stress	*Arabidopsis thaliana*	Provided a comprehensive view of AS under salt stress and revealed novel insights into the potential roles of AS in plant response to salt stress	[71]
Salinity	*Rice*	Candidate genes can be identified by QTL	[65]
Drought	Barley *(Hordeum spontaneum)*	Exploring the genomic basis of reproductive success under stress in wild progenitors with expected ecological and economic applications	[72]
Drought	Willow (paper-mulberry)	A core set of candidate genes encoding proteins with a putative function in drought response was identified	[73]
Salinity	Wild barley	Across many traits, QTLs that increased phenotypic values were identified	[74].
Salinity	Rice	Unveiled genomic regions/candidate genes regulating salinity stress tolerance in rice	[75]
Drought	Alfalfa (*Medicago sativa* L.)	Improved alfalfa cultivars with enhanced resistance to drought and salt stresses	[76]
Drought	Rice	Drought-induced alterations to DNA methylation that may influence epigenetics	[77]
Drought	Wheat	Thirty-seven of the significant marker-traits were detected under the drought-stressed condition	[67]
Drought	Wheat	Identified a QL on chromosome 4H	[78]

**Table 5:** Target gene-silencing techniques for improving crop variety under salinity and drought stresses.

Stresses	Crops	Silencing Gene	Major Findings	References
Cold, drought, salt stress	Rice	*OsNAC5*	RNAi lines became less tolerant of these stresses than control plants	[58]
Salinity	Arabidopsis	*sos1*	*thsos1*-RNAi lines of *Thellungiella* were highly salt-sensitive	[99]
Salinity	pepper	*CaATG8c*	The silencing of *CaATG8c* made pepper seedlings more sensitive to salt stress	[100]
Salinity	*Alternanthera philoxeroides*	*ApSI1*	Significantly decreased tolerance to salinity	[101]
Drought	*Alternanthera philoxeroides*	*ApDRI15*	Plants were more sensitive to drought stress than the control plants	[101]
Drought	Tomato	*SpMPK1, SpMPK2, and SpMPK3*	Reduced drought tolerance in tomato plants	[102]
Drought	wheat	*Era1* and *Sal1*	Played imperative roles in conferring drought tolerance	[103]
Drought, salt stress	Cotton	*GH3.17*	Enhanced drought and salt stress	[104]
Salinity	Cotton	*GhWRKY6*	Downregulation of *GhWRKY6* increased salt tolerance	[105]

**Table 6:** CRISPR/Cas9 technology-mediated improvements to plant tolerance to abiotic stress.

Target Genes	Crops	Target Stresses	References
*TaDREB2* and *TaERF3*	Wheat	Abiotic stress response	[118]
*ScNsLTP*	Sugarcane	Drought and chilling resistance	[119]
*MaAPS1* and *MaAPL3*	Banana	Cold and salt	[120]
*MeKUP*	Cassava	Salt, osmosis, cold, and drought resistance	[121]
*MeMAPKK*	Cassava	Drought resistance	[122]
*GhPIN1–3* and *GhPIN2*	Cotton	Drought resistance	[123]
*GhRDL1*	Cotton	Drought resistance	[124]
*CpDreb2*	Papaya	Drought, heat, and cold resistance	[125]
*OsDST*	Indica mega rice cultivar	Salt and Drought	[126]
*SlNPR1*	Tomato	Drought	[127]
*Leaf1,2*	Rice	Drought	[128]

**Table 7:** Marker-assisted breeding results for improving crop quality under salinity and drought stresses.

Stresses	Crops	Target Genes	Major Effect/Finding	References
Drought and Salt	Cotton		Significant associations between polymorphic markers and drought and salt tolerant traits were observed using the general linear model (GLM)	[48]
Salinity	Rice	*RM223*	Transferring genes from one variety to another and their use in MAS	[134]
Drought	Rice		Developed high-yielding rice cultivars suitable for water-limited environments through marker-assisted breeding	[135]
Salinity	Rice	*NAL1*	High yield through optimizing transportation efficiency of photosynthetic products by marker-assisted selection	[136]
Drought and flood	Rice		Developed high-yielding drought- and submergence-tolerant rice varieties using marker-assisted introgression	[25]
Drought	Rice		Provided a higher yield advantage	[137]
Drought	maize		Improved grain yield under drought stress conditions	[138]
Drought and salt	Wheat	TaCRT-D	Increased plant stress tolerance and the functional markers of TaCRT-D for marker-assisted selection in wheat breeding	[139]
Salinity	Rice		Developed new salt-tolerant rice germplasm using speed-breeding	[140]
Drought	Rice		Stimulated 10–36% higher yield among different inbred lines	[141]

**Table 8:** Characterized genes involved in abiotic stress tolerance through ROS regulation in crops.

Genes	Origin	Transformation Receptor	Protein Function	Major Functions	Signaling Hormone	Approaches Used	References
*GhMKK1*	*G. hirsutum*	*N. benthamiana*	MAPKK	Influences oxidative, ROS scavenging, salt and drought tolerance	Abscisic acid (ABA)	Reverse genetics	[147]
*DSM1*	*O. sativa*	*O. sativa*	MAPKKK	Influences oxidative, ROS scavenging, drought tolerance	ABA	RNA interference, Reverse genetics	[148]
*DSM2*	*O. sativa*	*O. sativa*	MAPKKK	Influences oxidative, ROS scavenging, drought tolerance	ABA	RNA interference, Reverse genetics	[149]
*MEKK1*	*Arabidopsis*	*Arabidopsis*	MAPKKK	Influences oxidative, ROS scavenging, abiotic stress tolerance	ABA	Reverse genetics	[150]
*GhMAPKKK49*	*G. hirsutum*	*G. hirsutum*	MAPKKK	ROS scavenging, salt, drought, and wounding stresses	ABA, gibberellins (GB), methyl jasmonate (JA), salicylic acid (SA), 6-benzyl amino purine, a-naphthyl acetic acid, and ethylene (ET)	Transcriptome	[151]
*MKK1, MKK2,* *MKK6*	*Arabidopsis*		MAPKK	Stimulate oxidative, ROS scavenging, abiotic stresses	SA	RNA interference	[150,152]
*OsCPK4*	*O. sativa*	*O. sativa*	Calcium-dependent protein kinase	ROS scavenging, drought, and salt stress	SA	Reverse genetics	[153]
*OsCPK12*	*O. sativa*	*O. sativa*	Calcium-dependent protein kinase	ROS scavenging, influences oxidative salt stress	ABA	RNA interference, Reverse genetics	[154]
*SiCDPK24*	*Setaria italica*	*Arabidopsis*	Calcium-dependent protein kinase	ROS scavenging, drought stress	ABA	Reverse genetics	[155]
*TaCIPK29*	*T. aestivum*	*N. benthamiana*	CBL-interacting protein	ROS scavenging, salt stress	ABA and ET	Reverse genetics	[156]
*MdCIPK6L*	Apple	*Arabidopsis*	CBL-interacting protein kinase	ROS scavenging, salt, osmotic/drought and chilling stresses	ABA	Reverse genetics	[157]
*MdSOS2L1*	Apple	tomato	CBL-interacting protein kinase	ROS scavenging, salt stresses	ABA	Reverse genetics	[158]
*AtCIPK5*	*Arachis diogoi*	*Arabidopsis*	CBL-interacting protein kinase	Salt and osmotic stress tolerance	NA	Reverse genetics	[159]
*SIT1*	*O. sativa*	*O. sativa*	Lectin receptor-like kinase	ROS production, salt sensitivity	ET	Reverse genetics	[160]
*OsMPK6*	*O. sativa*	*O. sativa*	MAPK	ROS scavenging, salt stresses	SA	RNA interference	[161]
*ZmMPKL1*	*Zea mays*	*Zea mays*	MAPK	ROS production, drought sensitivity	ABA	CRISPR/Cas9, Reverse genetics	[162]
*MeMAPK*	*Cassava*	*NA*	MAPK	osmotic, salt, cold, oxidative stressors	ABA	Transcriptome	[163]
*ZmMKK3*	*Zea mays*	*N. benthamiana*	MAPK	ROS scavenging, osmotic tolerance	ABA	Reverse genetics	[164]
*OsPP18*	*O. sativa*	*O. sativa*	Protein phosphatase 2C	ROS scavenging, drought and oxidative stress	ABA	RNA interference, Reverse genetics	[165]
*DST*	*O. sativa*	*O. sativa*	zinc finger C2H2	ROS scavenging, drought and salt stress	Cytokinins	QTL, RNA interference, Reverse genetics	[166,167]
*ZFP36*	*O. sativa*	*O. sativa*	zinc finger C2H2	ROS scavenging, stress and oxidative stress	ABA	RNA interference, Reverse genetics	[168]
*OsTZF1*	*O. sativa*	*O. sativa*	Zinc Finger Protein CCCH	ROS scavenging, drought, high-salt stress	ABA	RNA interference	[169]
*OsWRKY30*	*O. sativa*	*O. sativa*	WRKY	ROS scavenging, drought tolerance	SA	Reverse genetics	[170]
*GhWRKY6*	*G. hirsutum*	*Arabidopsis*	WRKY	ROS production, drought and salt stress	ABA	Transcriptome, VIGS, Reverse genetics	[105]
*EcNAC1*	*Helianthus annus*	*Helianthus annus*	NAC	ROS scavenging, salt stress	ABA	Reverse genetics	[171]
*NTL4*	*Arabidopsis*	*Arabidopsis*	NAC	ROS production, drought stress	ABA	RNA interference, Reverse genetics	[172]
*EcbHLH57*	*Eleusine coracana*	*N. benthamiana*	bHLH	ROS scavenging, salt, oxidative and drought stress	ABA	Reverse genetics	[173]
*JERF3*	*O. sativa*	*O. sativa*	Ethylene response factor (ERF)	Drought and osmotic stress	ET	Reverse genetics	[174]
*MnSOD*	*N. plumbaginifolia*	*M. sativa*	MnSOD	ROS scavenging drought stress	NA	Reverse genetics	[175]
*OsAPX2*	*Medicago sativa*	*Medicago sativa*	APX	ROS scavenging, salt tolerance	ABA	Reverse genetics	[176]
*PgGPX*	*Pennisetum glaucum*	*O. sativa*	GPX	ROS scavenging, salinity and drought stress	SA	Reverse genetics	[177]
*MsALR*	*M. sativa*	*N. benthamiana*	NADPH-dependent aldose/aldehyde reductase	Antioxidative metabolism, drought and oxidative stress	NA	Reverse genetics	[178]
*AtMIOX4*	*Arabidopsis*	*Arabidopsis*	MIOX	ROS scavenging, salt tolerance	ABA	Reverse genetics	[179]
*MtPP2C*	*Medicago truncatula*	NA	PP2C	ROS scavenging, drought and cold stress responses	ABA	Transcriptome	[180]
*OsAHL1*	*O. sativa*	*O. sativa*	AHL	ROS scavenging, drought resistance	ABA, SA	GWAS, Reverse genetics	[181]
*OsHK3*	*O. sativa*	NA	HK	ROS scavenging, salinity and drought stress	ABA	RNA interference	[182]
*IcSRO1*	*Ipomoea cairica*	*Arabidopsis*	SRO	ROS scavenging, salt and drought tolerance	ABA	Transcriptome, Reverse genetics	[183]
*OsCATB*	*O. sativa*	*O. sativa*	CATB	ROS production, drought stress	ABA	Transcriptome	[184]
*RBOHH*	*O. sativa*	*O. sativa*	NADPH Oxidase	ROS production, drought stress	ET	CRISPR/Cas9, Reverse genetics	[185]

**Table 9:** A summary of identified genes and their processes under salinity and drought stresses.

Functional Category	List of Genes	Type of Stress	Biological Function and Signaling Pathway	Tools Used	References
Protein kinase					
MAPKKK	*MEKK1, MEKK2,* *MEKK3, MEKK4,* *MAPKKK18, GhMAP3K40* *, OsMAPKKK63* *, GhMAPKKK49* *DSM1, DSM2*	Influences oxidative, abiotic, and biotic stress.	Growth and development; ABA	RNA interference, reverse genetics	[150,186,187]
MAPKK	*MKK1, MKK2,* *MKK6, GhMKK1,*	Influences oxidative, salt and drought	Growth and development; SA	Transcriptome, reverse genetics	[150,188]
	*MKK3, GhMKK3*	Influences oxidative, salt, and drought stresses	Growth and development; SA	RNA interference, reverse genetics	
	*MKK4, MKK5 GhMKK4,* *GhMKK5,*	Influences oxidative, drought	Growth and development; JA	RNA interference, reverse genetics	
	*MKK7, MKK8, MKK9,* *MKK10, RhMKK9,* *GhMKK9, ZmMKK10*	Salt and/or drought	Growth and development; ET	Reverse genetics	
	*VvMKK2, VvMKK4*	Influences oxidative, salt, and drought	Growth and development; SA	Reverse genetics	[177]
MAPK	*MPK3, MPK6, MPK10* *OsMPK6, ZmMPK3, RhMPK6,* *ZmMPK6-2, OsMPK3,* *ZmMPK3*	Influences oxidative, abiotic, and biotic stresses	Cell cycle regulation, cell division; JA and ET	RNA interference, reverse genetics	[150,189]
	*MPK4, MPK5, MPK11, MPK12, MPK13, OsMPK4ZmMPK4-1,* *OsMPK5, OsMPK5, ZmMPK5*	Influences oxidative, salt, and/or drought	Cell cycle regulation; SA	RNA interference, reverse genetics	[150]
	*MPK1, MPK2, MPK7,* *MPK14, ZmMPK7,* *OsMPK2AtMPK7,* *OsMPK7, GhMPK7*	Influences oxidative, salt, drought	Circadian-rhythm-regulated; JA, SA	RNA interference, reverse genetics	[150]
	*MPK8, MPK9,* *MPK15/16/17/18/19/20* *GhMPK17, ZmMPK17*	Influences oxidative, salt, drought	Cell cycle regulation; JA	RNA interference, reverse genetics	[161]
CDPK	*OsCPK4* *OsCPK12* *SiCDPK24* *FaCDPK4, FaCDPK11* *StCDPK3, StCDPK23*	Influences oxidative, salt, drought	Responses to developmental and environmental cues; SA, ABA	Transcriptome, RNA interference, reverse genetics	[190]
CIPK	*TaCIPK29* *MdCIPK6L* *MdSOS2L1* *AtCIPK5*	ROS scavenging, salt and osmotic stress tolerance	tissue and organ development; ABA	Reverse genetics	[167,168,169,170]
Transcription factor					
bZIP	*ABF3, BF4* *ABF3, ABF4* *FtbZIP5, PtrABF* *OsbZIP23,* *OsbZIP12,* *OsbZIP71, OsbZIP46* *OsbZIP72, ZmbZIP4* *OsbZIP62, TabZIP*	Salt, drought	Light signaling, seed maturation, flower development; ABA	Transcriptome, RNA interference reverse genetics	[191,192,193,194,195,196,197,198,199,200]
bHLH	*MYC2, AtbHLH17, AtbHLH68, AtbHLH122, FtbHLH2, FtbHLH3, PebHLH35, OsbHLH148*	Salt, drought	Growth, development, response to various stresses; JA, ABA	Transcriptome, RNA interference, reverse genetics	[201,202,203,204,205,206,207,208]
NAC	*ANAC019, ANAC055,* *ANAC072, ANAC042,* *TaNAC29, OsNAC6,* *OsNAC5, OsNAC9,* *OsNAC10, TaRNAC1,* *GmNAC109,* *CaNAC035*	Salt, drought	Plant growth and development range from the formation of shoot apical meristem, floral organ development, reproduction, lateral shoot development; ABA	Transcriptome, RNA interference, reverse genetics	[209,210,211,212,213,214,215,216,217,218,219]
AP2/ERF	*CBF1, CBF2, CBF3,* *AtERF53, AtERF74,* *AhDREB1, OsDREB1, OsEREBP1, OsERF7,* *GmERF3, ZmDREB2A,* *SlERF5*	Salt, drought	Regulation of plant growth and development; ABA	Transcriptome, RNA interference reverse genetics	[220,221,222,223,224,225,226,227,228,229,230]
MYB	*AtMYB44, AtMYB96,* *AtMYB20, OsMYB4,* *OsMYB6, OsMYB48-1,* *OsMYB91, GmMYB76,* *GmMYB92, GmMYB177*	Abiotic stresses	Circadian rhythm, regulation of primary and secondary metabolism; ABA, JA	Transcriptome, RNA interference reverse genetics	[231,232,233,234,235,236,237,238,239]
WRKY	*OsWRKY11, OsWRKY45, TaWRKY1, TaWRKY33,* *cWRKY023, ZmWRKY33, VvWRKY2*	Salt, drought	Growth and development; ABA	Transcriptome, RNA interference reverse genetics	[240,241,242,243,244,245]
ROS-scavenging					
SOD	*FSD1, FSD2, FSD3* *CSD1, CSD2, CSD3* *MSD1*	Salt, drought	Antioxidant defense against oxidative stress; ABA	RNA interference, Reverse genetics	[246]
	*CmSOD*	Oxidative stress	ABA	reverse genetics	[247]
	*CsSOD*	Drought	JA and gibberellin (GA3)	Transcriptome	[248]
APX	*APX1-APX7*	Salt and or drought	Growth regulation; ABA	Transcriptome	[246]
	*OsAPX1, OsAPX2*	Oxidative, Salt, drought	ABA	Transcriptome	[249]
	*OsAPX3, OsAPX4*	Salt and drought	ABA	Transcriptome	[249]
	*OsAPX5, OsAPX6 and OsAPX7*	salinity	ABA	Transcriptome	[250]
	*AgAPX1*	Drought	NA	Reverse genetics	[251]
	*TbAPX*	Salt	ABA	Reverse genetics	[252]
	*CytAPX*	Salt	ABA	Reverse genetics	[253]
CAT	*CAT2, CAT3, ScCAT1*	Salt and/or drought	ABA	Transcriptome	[254]
	*HuCAT3*	Salt and drought	NA	Transcriptome	[255]
	VsCat	Salt	Salt	CRISPR/C as9	[198]
	*CsCAT3*	Tolerance to heat, cold, salinity and osmotic condition	ABA	Transcriptome, Reverse genetics	[256]
GPX	*GPX1, GPX2, GPX5, GPX6* *and GPX7*	Abiotic stress	Plant development, multiple signaling pathways	Transcriptome	[257,258]
	*PgGPx*	Salinity and Drought	NA	Reverse genetics	[177]
	*ClGPX*	Salinity and Drought	ABA	Transcriptome	[258]
	*NnGPX*	Salt	NA	Reverse genetics	[259]
	*OsGPX5*	Salt	ABA	Transcriptome, RNA interference	[260]
MDHAR	*MDAR2-4*	Salt	Stress protection; ABA	Transcriptome	[256]
	*AeMDHAR*	Salt	NA	Reverse genetics	[261]
	*AtMDAR1*	Ozone, salt and drought stress	ABA	Reverse genetics	[262]
	*TrMDHAR*	salt	ABA	Transcriptome	[261]
DHAR	*SlDHAR1 and SlDHAR2*	salt	Stress protection; NA	Transcriptome	[263]
	*DHAR1 and DHAR3*	Salt	ABA	Transcriptome	[256]
	*LcDHAR*	Salt and drought	NA	Transcriptome, Reverse genetics	[264]
	*TrDHAR*	Salt	ABA	Transcriptome	[265]

## Data Availability

Most of the recorded data are available in the tables and figures of the manuscript.
